# Three-year observational study of immediate post-abortion insertion versus menstrual insertion of etonogestrel contraceptive implant

**DOI:** 10.1186/s12905-021-01542-8

**Published:** 2021-12-29

**Authors:** Wanren Zheng, Yibo Tang, Chunfen Wang, Xiaocen Niu, Zhida Qian, Lili Huang

**Affiliations:** 1 Women’s Hospital, Zhejiang University School of Medicine, 1 Xueshi Road, Hangzhou, 310006 Zhejiang Province People’s Republic of China; 2 Maternal and Child Health Institute of Zhejiang, Linan City, People’s Republic of China

**Keywords:** Etonogestrel implant, Vaginal bleeding pattern, Abortion, Contraception

## Abstract

**Objective:**

This study aimed to estimate the difference in vaginal bleeding pattern, discontinuation rate, and satisfaction between immediate after abortion and menstrual insertions of etonogestrel contraceptive implants.

**Study design:**

Between May 2013 and November 2015, 66 women were recruited in the abortion group who selected etonogestrel implants as their contraceptive immediately after induced abortion. 84 women who underwent the placement of the etonogestrel implant during their menstrual period were enrolled as the menstrual group. The two groups participated in 3-year follow-up outpatient visits at 1, 6, 12, 24, and 36 months after implantation. The vaginal bleeding pattern, discontinuation rate, satisfaction rate were recorded and compared.

**Results:**

No woman had pregnancy over the study period of 3 years. The incidence of amenorrhea/infrequent bleeding did not differ between the two groups after 12, 24, and 36 months of implantation (53.0% vs. 58.4%, 47.8% vs. 51.6%, and 48.6% vs. 55.6%, respectively). In the abortion group, the incidences of frequent/prolonged bleeding were 15.1%, 32.6%, and 27.0% after 12, 24, and 36 months of implantation, respectively, while the other group showed 27.3%, 25.8%, and 20.4%, respectively. After 12 and 24 months, the continuation use rates were 69.7% and 56.1% in the abortion group and 73.8% and 64.2% in the menstrual group. The 12-month satisfaction rate between abortion group and menstrual group was 69.6% versus 72.6%. Statistical analyses show that there was no difference in vaginal bleeding pattern, discontinuation rate or satisfaction between the two groups.

**Conclusions:**

Immediately post-abortion may be also a favorable time to undergo etonogestrel implantation.

## Introduction

The lack of adequate and effective contraceptive measures leads to approximately 46 million women worldwide undergoing surgical abortion. Almost 95% of induced abortion occurred in developing countries [1]. How to provide effective contraceptive methods to women after induced abortion and protect women’s reproductive health is a public health challenge for almost all governments and institutions worldwide [2]. In recent years, prospective studies in Eastern European countries have found that surgical abortion rates significantly decreased with the use of effective contraceptives [3, 4].

Unintended pregnancy is the most common reason for induced abortion. Correct and consistent use of effective contraceptive methods after abortion is an effective method to prevent unintended pregnancy and reduce the repeated abortion rate. The World Health Organization (WHO) recommends the use of subcutaneous implants immediately after abortion [5]. Etonogestrel implantation (Implantation), which is a single pure progestin-releasing subcutaneous rod, is a 3-year effective and reversible contraceptive. It was approved by the US Food and Drug Administration in 2006, and it is now available in more than 40 countries. In China, etonogestrel implantation is a very novel contraceptive method to both clinicians and women. Chinese clinicians currently have no clinical experience inserting the etonogestrel implant immediately post-abortion. Some clinicians wonder if placement immediately post-abortion may have a higher termination rate than the normal menstrual placement. In this study, the safety and acceptability of placement were evaluated by observing the vaginal bleeding pattern, continuation use rate, and satisfaction rate among women who received etonogestrel implantation immediately after abortion.

## Materials and methods

After approval was obtained from the Women’s Hospital School of Medicine Zhejiang University Institutional Review Board, 150 women who underwent etonogestrel implantation in the institution from May 2013 to November 2015 were enrolled in this study. In total, 66 women who chose to have the etonogestrel implant placed immediately after surgical abortion were recruited in the abortion group, and 84 women who chose to have the etonogestrel implant placed in menstrual d1–d5 were enrolled in the menstrual group.

Pre-operation included counselling ultrasonography, routine blood work, blood biochemistry, coagulation function, cervical cytology, gynaecological examination, and breast examination. Inclusion criteria: age 20–44 years old; menstrual cycle 28 ± 7 days; menstrual period 3–7 days; normal menstrual bleeding; gestational days of 42–63 with no abnormal bleeding during pregnancy. Exclusion criteria: Irregular vaginal bleeding of unknown cause; breast cancer patients; heavy menstrual bleeding; coagulation disorders; with or have suffered from ischaemic heart disease, history of cerebrovascular accidents, liver tumours, severe liver dysfunction, acute deep venous thrombosis, and pulmonary embolism. All participants signed an informed consent. Their telephone numbers, emails, WeChat and addresses were collected for follow-up. They were required to go to the clinic for follow-up at 1, 6, 12, 24, and 36 months after implantation.

The etonogestrel implants were inserted in accordance with the manufacturer’s instructions by trained experienced surgeons in the family planning department.

The contraceptive effect, vaginal bleeding pattern changes (daily menstrual diary records), continued use rate, and reasons for removal were recorded by participants. The participants completed the satisfaction questionnaire 12 months after the implantation (removal was included in “not satisfied”).

The SPSS 19.0 software was used for statistical analysis. Measurement data are expressed as the mean ± standard deviation. The mean comparisons between the two groups were tested using a t-test. The rate comparisons between the two groups were performed using the chi-square test. The unadjusted hazard ratio (HR) and 95% confidence interval (CI) was calculated for a crude assessment of the relative risk of vaginal bleeding pattern changes after implantation using the menstrual group as the reference group. Statistical significance was defined as *P* < 0.05.

## Results

### Participant characteristics

In total, 150 women were recruited into the study. Among them, 66 had the etonogestrel implant immediately post-abortion. Their detailed characteristics are shown in Table 1.

**Table 1:** Basic characteristics of two groups

Group	Number	Age (year)	Weight (kg)	Gravidity	Abortions
Abortion	66	20–43 (30.44 ± 5.55)	40–75 (53.73 ± 6.99)	0–10 (3.79 ± 2.20)	0–8 (2.67 ± 2.09)
Menstrual	84	21–42 (32.32±4.94)	42–78 (54.48±6.82)	0–9 (3.40±1.71)	0–7 (2.32±1.54)

### Vaginal bleeding pattern changes after the etonogestrel implantation between the two groups

The vaginal bleeding pattern was based on a WHO study with a 90-day reference period [6]. It was defined as (1) amenorrhea: no bleeding and/or blood loss within 90 days; (2) infrequent bleeding: fewer than 2 bleeding/spotting episodes within 90 days; (3) frequent bleeding: more than 4 bleeding/spotting episodes within 90 days; (4) prolonged bleeding: at least one bleeding/spotting episode lasting 14 days or more. No significant difference was noted in the variation of vaginal bleeding pattern between the two groups. Table 2 shows the details.

**Table 2:** Variation in vaginal bleeding pattern in the two groups

	Abortion group	Menstrual group	χ^2^/*P* value	Relative risk(95% CI)
Bleeding pattern at 12 months after insertion	n = 66	n = 84	0.272/0.602	1.2 (0.49–2.92)
Amenorrhea/infrequent bleeding	19/16 (53.0)	34/15 (58.4)		
Frequent/prolonged bleeding	8/12 (15.1)	5/18 (27.3)		
Bleeding pattern at 24 months after insertion	n = 46	n = 62	0.468/0.494	0.83 (0.32–2.13)
Amenorrhea/infrequent bleeding	6/16 (47.8)	14/18 (51.6)		
Frequent/prolonged bleeding	7/8 (32.6)	4/12 (25.8)		
Bleeding pattern at 36 months after insertion	n = 37	n = 54	0.620/0.431	1.01 (0.38–2.6)
Amenorrhea/infrequent bleeding	4/14 (48.6)	10/20 (55.6)		
Frequent/prolonged bleeding	5/5 (27.0)	4/7 (20.4)		

### Discontinuation of etonogestrel implant between two groups

Twenty and 22 women in the abortion and menstrual groups, respectively, removed the etonogestrel implant after 12 months, and the continuation ratewas 69.7% (46/66) and 73.8% (62/84), respectively. The 24-month continuation rate declined to 56.1% (29 removed) in the abortion group and 64.3% (54/84) in the other group. The main reasons for discontinuation were bleeding, weight gain, or planned pregnancy. “Migration” means movement from the site of insertion. The 36-month continuation rates were 39.4% (26/66) in the abortion group and 47.6% (40/84) in the menstrual group, and they did not significantly differ. Table 3 enumerates the details.

**Table 3:** Discontinuation at 12, 24, 36 months after placement

Removal reasons	1–12 months		12–24 months		24–36 months	
	Abortion	Menstrual	Abortion	Menstrual	Abortion	Menstrual
Bleeding	10	12	5	4	8	6
Amenorrhea	3	5	1	1	2	0
Weight gain	4	3	1	2	1	1
Planned pregnancy	1	1	2	1	0	5
Migration	1	0	0	0	0	0
Others	1	1	0	0	0	2
Total	20	22	9	8	11	14
	χ^2^ = 0.310, *P* = 0.578		χ^2^ = 0.884, *P* = 0.347		χ^2^ = 0.159, *P* =0.690	

### Twelve-month satisfaction rate in two groups

The 12-month satisfaction rates were 69.6% in the abortion group and 72.6% in the menstrual group. No significant difference was found between the two groups (*P* > 0.05), as shown in Table 4.

**Table 4:** Twelve-month satisfaction rate between two groups

Group	Very satisfied	Satisfied	General	Not satisfied	Total
Post-abortion	2 (3.0)	36 (54.5)	8 (12.1)	20 (30.4)	66
Menstrual	6 (7.1)	46 (54.8)	9 (10.7)	23 (27.4)	84
χ^2^ = 1.347,*P* = 0.718					

In this study, all 150 women had successful contraception during the 3-year period of etonogestrel implant placement.

## Discussion

The etonogestrel implant is a long-acting reversible contraceptive method, and it is safer, more efficient, simpler, and more cost effective than oral contraceptives [7]. The etonogestrel implant mainly achieves contraception by inhibiting ovulation. Once the implant is inserted, compliance is ensured, and its effectiveness is less affected by the user. Many large-scale clinical trials have confirmed that the incidence of accidental pregnancy in the first year of subcutaneous embedding of contraceptives is < 0.05% [8].

Long-acting reversible contraceptive (LARC) provision is an important part of reducing the unintended pregnancy rates after induced abortion. In present study, there was no significant difference in vaginal bleeding patterns, continuation rates, or satisfaction rates among women who had etonogestrel implants placed immediately after abortion or during their menstrual period. Thus, the placement of the etonogestrel implant immediately after abortion is a suitable period.Ovulation may resume within 2 weeks after induced abortion.Unexpected pregnancy can reoccur in a short time. The etonogestrel implant can be used to prevent contraception immediately after abortion and reduce repeated abortion.

In this study, 66 women had etonogestrel implants placed immediately post-abortion. No significant difference was observed between abortion and menstrual groups in terms of the vaginal bleeding pattern, continuation use rate, or 12-month satisfaction. Therefore, the implementation of the etonogestrel contraceptive implant immediately post-abortion provides convenience for women, helps avoid repeated pregnancy, reduces the risk of unintended pregnancy, and protects women’s reproductive function.

We found that the 12-month satisfaction questionnaire showed no difference between the two groups, and women were satisfied with the two different implantation times. Whether amenorrhea is present after implantation did not affect satisfaction. However, frequent/prolonged bleeding after implantation affected the satisfaction of the recipients. In particular, spot bleeding may last longer alter the original menstrual cycle and cause inconvenience in the daily and sexual lives of women. Women were informed of possible side effects ,such as weight gain, hyperpigmentation, acne, distending breast pain. But when they met some of those side effects personally, they may still find it difficult to accept them.

This study also found that the continuation rate of the two groups notably differed from the results of foreign studies [9]. The present study indicates that women in China have low acceptability of etonogestrel implants. Possible reasons are different cultural backgrounds, pressure and influence of the traditional family planning policy, lack of knowledge regarding new contraceptive methods. These participants possibly removed the implant when they experienced dissatisfaction because of low tolerance. Common reasons for early discontinuation were vaginal bleeding, amenorrhea, and hormone-related side effects.

In summary, the etonogestrel implant has a reliable contraceptive effect with no major adverse reactions. Immediate placement post-abortion may be a suitable time.

## Data Availability

The datasets used and/or analysed during the current study are available from the corresponding author upon reasonable request.
